# Evidence-Based Weight Management for Fertility Preservation in Endometrial Cancer Patients: Developing a Complex Intervention Based on the Medical Research Council Framework

**DOI:** 10.3390/healthcare13131623

**Published:** 2025-07-07

**Authors:** Jingjing Gong, Yiqian Chen, Yongli Wang, Yuanyuan Gong, Dandan Yang, Xiaodan Li

**Affiliations:** 1 Department of Obstetrics and Gynecology, Peking University People’s Hospital, Beijing 100044, China; gongjingjing@pkuph.edu.cn (J.G.); wangyongli@pkuph.edu.cn (Y.W.); gongyuanyuan@pkuph.edu.cn (Y.G.); yangdandan@pkuph.edu.cn (D.Y.); 2 Department of Nursing, Beijing Health Vocational College, Beijing 101101, China; chenyiqian@wjw.beijing.gov.cn

**Keywords:** fertility-sparing treatment, weight management, complex intervention, midwifery

## Abstract

**Background/Objectives**: This study aims to develop a standardized weight management intervention program for patients with endometrial cancer (EC) undergoing fertility preservation treatment and to provide a scientific foundation for midwives to implement weight management initiatives in the domains of oncology and reproduction. **Methods**: The weight management intervention program for patients with EC undergoing fertility preservation treatment was crafted following the directives of the Medical Research Council framework for developing and assessing complex interventions and the World Health Organization handbook for guideline development. The development process encompassed four distinct stages: (1) establishing the intervention development group, (2) identifying a theoretical basis and forming a content framework, (3) gathering and synthesizing evidence, and (4) refining and modeling the practice program. **Results**: The ultimate weight management program consisted of 6 primary, 18 secondary, and 53 tertiary items. Through two rounds of Delphi consultation, a response rate of 100% was attained, with an expert authority coefficient of 0.83. **Conclusions**: The developed intervention demonstrates scientific robustness and clinical feasibility, presenting a structured methodology for weight management for EC patients undergoing fertility preservation therapy.

## 1. Introduction

Endometrial cancer (EC) is a common disease in oncofertility and ranks as the sixth most common cancer in women, with over 427,000 newly diagnosed cases worldwide in 2022 [1]. Currently, total hysterectomy remains the standard treatment [2]. In recent years, there has been a noticeable shift towards younger age groups in EC cases. More than 5% of the diagnosed EC patients fall within the 35–44 age bracket, and 2% are aged between 20 and 24 [3]. Preserving female fertility has become a significant focus in clinical practice, particularly for young patients with well-differentiated endometrial adenocarcinoma and atypical endometrial hyperplasia. The primary approach involves continuous high-dose progesterone therapy to induce endometrial regression and establish a fertility window for potential pregnancy [4]. Despite a remission rate of up to 90% following fertility-preserving treatment for EC, challenges such as progesterone resistance and recurrence persist, with obesity emerging as a significant contributing factor. Park et al.’s retrospective study [5] found that a BMI ≥ 25 kg/m^2^ was associated with poor response rates and higher recurrence. Barr et al.’s randomized controlled trial [6] demonstrated that obese women with endometrial atypical hyperplasia or EC may experience improved tumor outcomes through interventions like bariatric surgery or low-calorie diet-induced weight loss. Additionally, research indicates that individuals with a BMI < 25 kg/m^2^ exhibit a two-fold higher natural pregnancy rate compared to the obese cohort [7]. In a retrospective study conducted by Zhang et al. [8], it was observed that patients with EC in the weight loss group exhibited increased pregnancy rates and live birth rates. Obesity, as an independent risk factor for developing EC, can impact the effectiveness of tumor treatments [9]. These findings underscore the significance and necessity of weight management for EC patients undergoing fertility preservation treatments.

In the context of fertility preservation for patients with EC, multidisciplinary collaboration is essential. Among the key members of the multidisciplinary team, midwives play a pivotal role that encompasses not only weight management, fetal development assessment, and maternal health evaluation but also extends to providing psychosocial support and educational guidance. This comprehensive care approach spans the entirety of treatment and pregnancy. Midwives play a critical role in the early identification and intervention for high-risk patients, necessitating an expansion of their traditional perinatal care responsibilities to actively engage in oncofertility-related interventions. While current treatment guidelines and expert consensus documents from academic societies and professional organizations [2,10,11,12] emphasize the necessity of weight management in fertility preservation for EC patients, specific implementation strategies are lacking. This gap has prompted considerations regarding the expanded role of midwives in this field.

The objective of this study is to develop a comprehensive weight management intervention protocol utilizing the Medical Research Council (MRC) framework. This protocol aims to offer scientific substantiation to midwives involved in weight management for EC patients undergoing fertility preservation. The research seeks to establish a strong scientific basis for midwives to improve patient management and support comprehensively, enabling them to address weight-related challenges more effectively and improve fertility preservation outcomes in EC patients.

## 2. Materials and Methods

**Figure 1:**
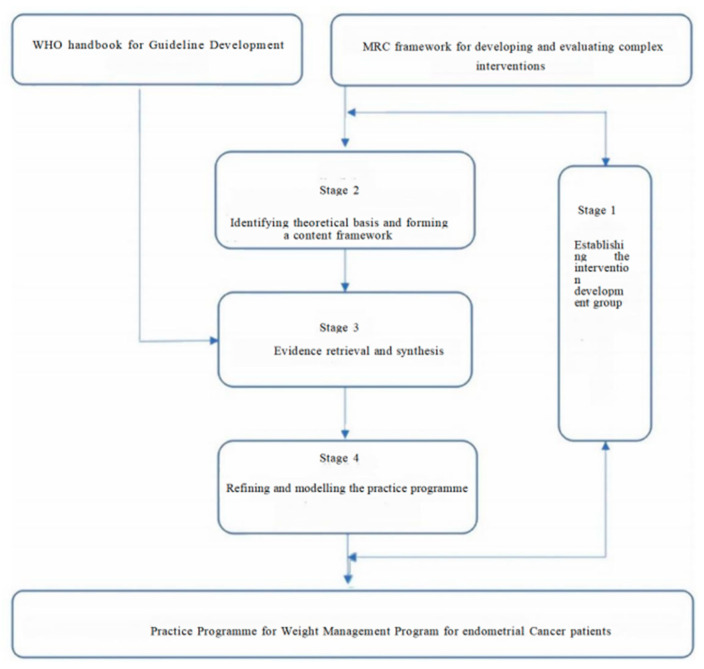
Diagram for the development process of the Practice Programme for Weight Management Program for endometrial Cancer patients.

This study adhered to the UK Medical Research Council (MRC) framework for developing complex interventions [13], integrating the Nutrition Care Process Model (NCPM) and the Health Belief Model (HBM) to guide the intervention design. The development process comprised four stages. Stage 1 involved establishing the intervention development team, which comprised a multidisciplinary group consisting of gynecologists, nutritionists, endocrinologists, and nursing experts, to ensure comprehensive expertise in weight management and fertility preservation. Stage 2 focused on theoretical framework and content development, where the NCPM and HBM were utilized to structure the intervention, emphasizing patient education, behavior change, and nutritional support. In Stage 3, a systematic literature review of guidelines and expert consensus and an evidence synthesis were conducted to identify the best practices in weight management for overweight and obese patients. Finally, Stage 4 consisted of Delphi expert consultation, which entailed two rounds of consultations with 16 experts to refine the intervention, ensuring its clinical relevance and feasibility (Figure 1).

### 2.1. Stage 1: Establishing the Intervention Development Group

Establishing a well-rounded intervention development team is crucial for creating a high-quality complex intervention. Following this principle, the team should be multidisciplinary, encompassing experts in obstetrics and gynecology, midwives, kinesiology, nutrition, evidence-based medicine, management, and relevant stakeholders. This team actively participated across the iterative development stages, culminating in the final intervention program.

### 2.2. Stage 2: Identifying Theoretical Basis and Forming the Content Framework of the Weight Management Program

The identification of a theoretical basis is a critical step in intervention development. This phase involves conducting a literature review and descriptive phenomenological research to select appropriate theoretical models that will form the basis for the content framework of the planned development. According to the literature review, three loops of the Nutrition Care Process and Model (NCPM) were selected [14] to construct the implementation steps of a weight management plan for patients with EC who require fertility preservation, utilizing the inner loops of the model. Furthermore, a descriptive qualitative study was carried out in the gynecology department of a tertiary hospital in Beijing to examine the factors impeding weight management in patients.

### 2.3. Stage 3: Evidence Retrieval and Synthesis

#### 2.3.1. Inclusion and Exclusion Criteria

Inclusion criteria: (1) Participants were overweight or obese individuals, either general patients or those with EC. (2) The study focused on weight management strategies for overweight and obese individuals, encompassing nutrition, exercise, behavioral intervention, etc. (3) Accepted research types included guidelines, standards, consensus statements, and norms. Exclusion criteria: (1) Participants were under 18 years old; (2) document types excluded were guide interpretation, conference abstracts, patent documents, drafts; (3) repeated publications; (4) non-Chinese and English literature.

#### 2.3.2. Search Strategy

The computerized search encompassed three English databases: Web of Science, SCOPUS, and PubMed, along with four Chinese databases: CNKI, Wanfang, China Science and Technology Journal Database, and China Biomedical Literature Database. Additionally, relevant guidelines from international and national sources were explored, such as the International Guide Collaboration Network, the National Guide Database of the United States, and Yimaitong. The Chinese search terms included “overweight and obesity”, “weight management/weight loss/weight loss/fat loss”, “nutrition”, “exercise”, “behavior”, and “lifestyle”, while English search terms included “overweight/obesity”, “weight management/weight loss/fat loss”, “nutrition”, “exercise”, and “lifestyle”. The search period spanned from the database’s establishment to March 2022.

#### 2.3.3. Data Analysis

The retrieved literature was imported into NoteExpress software V3.5.0.9054 and subsequently reviewed based on author, year, and title. Two researchers independently screened the literature meticulously, adhering strictly to the classification criteria, and extracted detailed information, including research title, publication date, publication institution, and research type. Any discrepancies between the researchers were resolved through discussion, with consultation from a third researcher if necessary. Concurrently, a literature analysis was conducted on the included studies, extracting subject content related to weight management, defining category dimensions, and completing frequency statistics. A summary was then compiled.

### 2.4. Stage 4: Refining and Modeling the Practice Program

Enhancing the intervention plan is a pivotal aspect of clinical practice, necessitating revisions to both the content and methodology of the weight management program. These revisions encompassed expert consultations and modeling processes to refine the intervention plan effectively.

#### 2.4.1. Expert Consultation

This study conducted a Delphi expert consultation to refine a draft weight management plan for EC patients. The panel consisted of 16 experts specializing in gynecological oncology, clinical nutrition, sports medicine, endocrinology, clinical nursing, nursing management, and related fields, including clinical diagnosis and treatment, nursing, and scientific research, from tertiary-grade A general hospitals or universities in Beijing and other locations. The selection criteria for experts included: (1) active involvement in clinical diagnosis, nursing, or scientific research related to gynecological oncology, clinical nutrition, sports medicine, clinical nursing, in third-level first-class hospitals or universities; (2) a minimum of 5 years of relevant work experience; (3) holding an intermediate or higher professional title; (4) possessing a Bachelor’s degree or higher; and (5) obtaining informed consent from all participating experts. Notably, members of the intervention development team were excluded from the panel to ensure the rigor and scientific accuracy of the plan’s revision process.

#### 2.4.2. Improvement Process

The expert questionnaire was formulated and distributed to eligible experts. It comprised three sections: ① Investigation description: This section provided an overview of the research background, purpose, and significance, along with instructions for completing the questionnaire. ② Weight management program: The questionnaire included 6 primary items, 18 secondary items, and 53 tertiary items outlining the weight management program for EC patients undergoing fertility preservation treatment. Experts utilized a Likert 5-point scale to assess the importance of each indicator, categorized as significant, important, moderately important, not necessary, and very unimportant. Ratings ranged from 1 to 5, corresponding to these categories. Additionally, the column “Modification opinions” section allowed experts to offer suggestions or modifications for each indicator. ③ Expert information: This section gathered general details about the experts, their familiarity with the questionnaire content, and the rationale behind their judgments. Following the collection of responses from the initial round of the questionnaire, a group discussion was conducted based on the analysis of the importance scores of the correspondence items and expert feedback. Entries were screened based on the expert opinions and statistical results, leading to modifications and additions for the development of the second round of the expert questionnaire. This revised questionnaire was then circulated to the experts for a second round of feedback using the same methodology. The consultation process concluded when there was alignment between the importance ratings and expert opinions regarding weight management programs for patients with EC undergoing fertility preservation treatment.

### 2.5. Ethical Considerations

This study received approval from the Ethics Review Committee (IRB) of Peking University People’s Hospital in 2022 (2022PHB366-001).

## 3. Results

### 3.1. Stage 1: Establishing the Intervention Development Group

The intervention panel consisted of nine specialists, including two gynecological oncologists, one midwife, two experts proficient in evidence-based research, one clinical nutrition expert, one kinesiology expert, one endocrinology expert, and one care management expert. Additionally, one patient undergoing fertility preservation therapy for EC was included in the panel. The tasks assigned to the intervention team were as follows: (1) establishing the theoretical basis and content framework for a weight management program for EC patients; (2) identifying and searching literature integration evidence; (3) drafting and revising the weight management plan for EC patients.

### 3.2. Stage 2: Identifying Theoretical Basis and Forming a Content Framework

**Figure 2:**
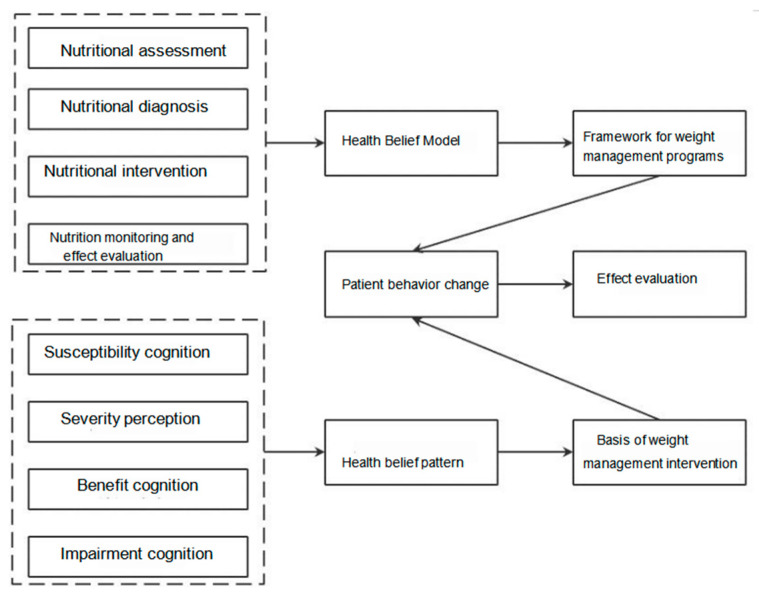
Theoretical framework diagram.

The Health Belief Model (HBM) is a standard nutrition education model developed by the American Academy of Dietetics and Nutrition in 2003, aimed at assisting nutrition professionals in managing dietary and healthcare concerns. In our weight management program, we commence from the standpoint of health belief establishment. The HBM serves as a robust theoretical foundation for devising weight management strategies tailored to patients with EC. Refer to Figure 2 for details.

Subsequently, a descriptive qualitative study was conducted to explore related factors impeding weight management in EC patients. A total of 15 EC patients receiving fertility preservation therapy participated in interviews to explore their perspectives on the link between obesity and EC, their weight loss experiences, and the type of assistance they desire from healthcare providers in managing their weight. Utilizing Colaizzi’s 7-step analysis, the qualitative data revealed three overarching themes and eight sub-themes. These encompassed information factors (obesity cognition, diet and nutrition, exercise, and information channels), safety factors (symptom management and program adjustment), and supporting factors (peer support and family support). Specifically, most patients pay insufficient attention to weight management due to constraints like time and financial limitations. Only a small number of patients seek nutrition clinics for professional weight loss guidance. Challenges related to information and safety could result in adverse reactions and exercise-related injuries during weight loss, significantly impacting their confidence in the weight management process. Patients expressed a desire for support from family and peers.

### 3.3. Stage 3: Evidence Retrieval and Synthesis

**Figure 3:**
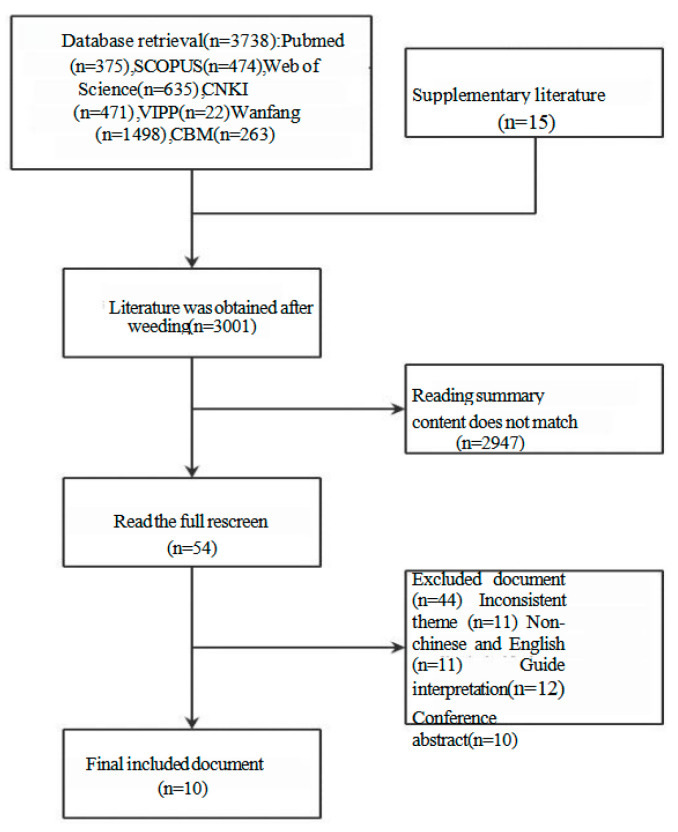
Literature screening process.

A total of 3753 articles were preliminarily searched, including 2254 in Chinese and 1499 in English. Following the screening process, 752 duplicate articles were removed through NoteExpress, 2947 papers were excluded based on title and abstract review, and 44 additional papers were excluded after full-text assessment. Ultimately, 10 papers were finally included, including 5 guidelines and 5 expert consensus. The literature screening process is illustrated in Figure 3, and the general characteristics of the included literature are presented in Table 1. Utilizing the nutritional program model, the management content concerning overweight and obesity in the literature was analyzed, leading to the identification of six key aspects. The findings of the literature analysis are detailed in Table 2.

### 3.4. Stage 4: Refining and Modeling the Practice Program

Based upon the established content framework and the results of the literature review, a draft weight management plan was developed. Relevant experts are invited to revise the weight management plan based on their extensive clinical expertise and profound theoretical understanding.

#### 3.4.1. Expert Consultation

Two rounds of expert consultations were conducted, involving 20 specialists who were invited to participate. Of the 16 specialists who agreed to take part in the survey, the composition included 6 gynecological oncology specialists, 4 clinical nutrition specialists, 2 care management specialists, 2 clinical nursing specialists, 1 sports medicine specialist, and 1 endocrinology medicine specialist. Upon acceptance of the invitation, questionnaires were distributed to the 16 experts to gather information on their personal characteristics and revision suggestions, with a 100% response rate for each round. The expert authority coefficient was calculated at 0.83. Following the initial expert consultation, Kendall’s W of the first-level, second-level, and third-level items was 0.500, 0.486, and 0.610, respectively. The coefficient of variation (CV) of the first-level items ranged from 0.00 to 0.08, for the second-level items from 0.00 to 0.17, and for the third-level items from 0.00 to 0.26. After the second round of consultation, Kendall’s W of the first-level, second-level, and third-level items was 0.333, 0.460, and 0.614, with corresponding CV ranges of 0.00–0.08, 0.00–0.17, and 0.00–0.25, respectively.

#### 3.4.2. Improve the Process Results

Following two rounds of expert consultations, adjustments, additions, and deletions were made to the program content. Expert feedback on all items converged toward consistency and stability. Consequently, the weight management program for EC patients undergoing fertility preservation treatment was refined and finalized, comprising 6 first-level items, 18 second-level items, and 53 third-level items.

## 4. Discussion

### 4.1. Literature Review Content Analysis

In the weight management program for EC patients, the establishment of a multidisciplinary diagnostic and treatment team comprising gynecologists, dietitians, nurses, and other healthcare professionals is essential to provide comprehensive support throughout the weight management process. Regarding screening, the guidelines and consensus documents reviewed all emphasized the use of BMI as a key indicator to identify overweight and obesity. However, there are variations in the evaluation criteria for overweight and obesity between different regions. In China, BMI ≥ 24 kg/m^2^ and <28 kg/m^2^ are considered overweight, and BMI ≥ 28 kg/m^2^ is considered obese [15,20,21,22,23,24]. Conversely, international standards consider BMI ≥ 25 kg/m^2^ and ≤29.9 kg/m^2^ as overweight, with BMI ≥ 30 kg/m^2^ indicating obesity [16,17,18,19]. NICE guidelines further classify obese people with BMI ≥ 30 kg/m^2^ into three levels [19]. Some guidelines also propose staging or classifying obesity based on BMI in conjunction with comorbidities or other parameters to guide weight management [20,23,24], although these criteria may vary across different guidelines and consensus documents. Different guidelines or consensus focus on distinct aspects when assessing overweight and obese patients, typically encompassing a history of overweight and obesity, complications and comorbidities, routine laboratory and instrument examination, energy intake and consumption, lifestyle and behavioral ability assessment, and psychological and quality of life assessment. In terms of making plans, most guidelines or consensus put forward weight loss goals. For instance, the Expert Consensus and Group Standard on Weight of Overweight or Obese People [24] suggests a weight loss percentage based on the stage of obesity without specifying the timeframe, while the AACE/ACE guidelines recommend different weight loss goals based on various obesity-related comorbidities [16]. “Guidelines for medical nutrition treatment of overweight/obesity in China (2021)” [15], Expert Consensus on Weight Management Procedures for Overweight or Obese People (2021), Expert Consensus on Body Quality Management Paths and Procedures for Overweight/Obese Infertility Patients in China [23], and EASO guidelines [18] all advocate for a weight loss target of 5% to 15% within a 3-to-6-month timeframe. Regarding the specific weight loss management of overweight and obese people, almost all guidelines or consensuses refer to the three cornerstones of diet, exercise, and behavioral intervention, alongside health education and psychological support. In terms of supervision and evaluation, five included studies proposed regular follow-up monitoring for weight management in obese individuals, detailing aspects such as follow-up duration, methodology, and content [15,18,19,20,24]. These insights hold significant instructive value for the development of weight management programs for EC patients.

### 4.2. Scientific and Feasibility Analysis of the Weight Management Program for EC Patients with Fertility Preservation

In the early stage of this study, an extensive and systematic literature review was conducted to explore and analyze relevant guidelines and consensus documents on weight management. This thorough examination provided a robust groundwork for constructing the content of the weight management plan tailored for EC patients seeking fertility preservation. The development of the initial plan was guided by a nutrition plan model, serving as the theoretical framework for structuring the overall weight management plan. Furthermore, the health education component of the project was designed based on the principles of the Health Belief Model, ensuring the creation of comprehensive and scientifically grounded health education materials related to weight management for EC patients with fertility preservation needs. To effectively address patient needs and develop a clinically meaningful weight management program, structured interviews were conducted with EC patients undergoing treatment in the initial phase. These interviews aimed to identify and consolidate the actual challenges faced by patients in managing their weight. Based on previous work, such as a literature review and analysis of obstructive factors, the initial draft of weight management for the fertility preservation treatment of EC patients was formed through group meeting discussions. Further, the Delphi expert inquiry method was adopted to ensure the scientific nature of the plan. Based on the specific patient needs, the weight management program entails delivering health education to address information factors, offering a scientifically sound weight management plan through personalized dietary, exercise, and behavioral recommendations, and overseeing and promoting patient weight management progress. Peer and professional support are provided through WeChat groups. Throughout the treatment cycle, patient diagnosis, treatment, and assessment are closely monitored, leveraging the weight management program’s impact on endometrial cancer patients undergoing fertility preservation. Online supervision and guidance are integrated with offline assessments, and hospital-based nursing management is coordinated with extended care outside the hospital to provide tailored professional support for weight management in EC patients.

### 4.3. Limitations and Outlook

The qualitative study involved interviewing only 15 patients from a single hospital, which restricts the generalizability of the findings. The weight management program in this study was tailored for overweight and obese patients and did not address the management of nursing patients with normal weight. Additionally, the application of weight management was limited to the phase of fertility preservation treatment for EC patients. Moving forward, weight management can be extended to pre-pregnancy, pregnancy, postpartum, and other stages after complete remission, potentially integrating molecular typing for precision care. At the policy level, there is potential to advocate for the integration of midwife-led weight management services into health insurance coverage and establish standardized service protocols. On a technical level, the development of a specialized AI dietitian system for endometrial cancer patients undergoing fertility preservation, incorporating electronic health records and wearable device data, could be explored. Implementation-wise, leveraging the expertise of midwives and conducting diverse health education initiatives on weight management are crucial. It is important to note that the effectiveness of these strategies in promoting weight loss in this specific population has not been assessed or implemented yet. The success of implementation will also depend on the variability and adherence to midwife training. The next step will involve evaluating the application effect of the weight management plan.

## 5. Conclusions

Through a comprehensive literature review and semi-structured interviews, this study has developed an initial weight management protocol for patients with EC undergoing fertility preservation therapy (see Table A1). Utilizing the Delphi expert correspondence method, each component of the program was evaluated for content and significance, leading to the finalization of the program’s draft. In the context of the “Healthy China 2030” initiative, addressing the treatment of EC patients with a focus on fertility preservation and sexual health management has emerged as a critical aspect of reproductive medicine. With the core concept of “whole person care”, midwives are tasked with elevating weight management from a singular metabolic intervention to a comprehensive initiative that encompasses physiological, psychological, and social relations. Leveraging technological advancements and social resources integration, the aim is to achieve the triple objective of “tumor remission–reproductive success–quality of life improvement”.

## Figures and Tables

**Table 1:** General information on the included literature.

Serial Number	Literature Name	Time	Issuing Authority	Literature Type
Guide1	Guidelines for medical nutrition treatment of overweight/obesity in China (2021) [15]	2021	Nutrition and Metabolic Management Branch of China International Exchange and Promotive Association for Medical and Health Care, Clinical Nutrition Branch of Chinese Nutrition Society, Chinese Diabetes Society, Chinese Society for Parenteral and Enteral Nutrition, Chinese Clinical Nutritionist Center of Chinese Medical Doctor Association	Evidence-based guidelines
Guide2	American Association of Clinical Endocrinologists and American College of Endocrinology Comprehensive Clinical Practice Guidelines for Medical Care of Patients with Obesity-Executive Summary [16]	2016	American Association of Clinical Endocrinologists	Evidence-based guidelines
Guide3	2013 AHA/ACC/TOS Guideline for the Management of Overweight and Obesity in Adults [17]	2014	ACC/AHA/TOS	Evidence-based guidelines
Guide4	European Guidelines for Obesity Management in Adults [18]	2015	EASO	Evidence-based guidelines
Guide5	Obesity: identification, assessment and management [19]	2014	NICE	Evidence-based guidelines
consensus1	Expert consensus on the procedure of body weight management among patients with overweight or obesity (2021) [20]	2021	Chinese Society of Health Management, Clinical Nutrition Branch of Chinese Nutrition Society, Medical Nutrition Industry Branch of the National Association of Health Industry and Enterprise Management, the Editorial Board of Chinese Journal of Health Management	Expert consensus
consensus2	Multidisciplinary Clinical Consensus on Obesity (2021) [21]	2021	Chinese Society of Endocrinology (CSE), Diabetes Society of China Association of Chinese Medicine (DSCACM), Chinese Society for Metabolic and Bariatric Surgery (CSMBS), Chinese Association of Research Hospitals (CSDBS)	Expert consensus
consensus3	Expert consensus on nutritional therapy for patients with EC [22]	2020	Chinses Society of Nutritional Oncology	Expert consensus
consensus4	Expert consensus on the weight management of overweight/obese infertility patients in China [23]	2020	Chinese Experts Consensus Group on the Body Quality Management Approach and Process for Overweight/Obese Infertility Patients.	Expert consensus
consensus5	Expert consensus & standard on weight management for overweight or obese people [24]	2018	Chinese Society of Health Management Chinese Nutrition Society Reproductive Medicine Branch of China International Exchange and Promotion Association for Medicine and Healthcare China Health Promotion Foundation Zhejiang Provincial Clinical Nutrition Center	Expert consensus

**Table 2:** Literature content analysis table.

Item	Frequency	Content
1. Establishing the intervention development group	6	Establish a multidisciplinary diagnosis and treatment team for obesity management [16,17,18,19,20,21]
2. Screening and evaluation	10	(1) Overweight and obese based on BMI [15,16,17,18,19,20,21,22,23,24] (2) Staging or classification of overweight and obesity based on BMI and comorbidities [20,23,24] (3) Patients’ history of overweight and obesity, complications and comorbidities, routine laboratory and instrument examination, energy intake and expenditure [15,16,18,20,24], lifestyle and behavioral ability assessment, psychological and quality of life assessment [15,18,19,21,24]
3. Make a plan	10	(1) Set weight loss goals [18,20,22,24], 3 to 6 months of weight loss of 5% to 15% [18,20,24] (2) Develop an individualized diet and exercise program [16,17,18,19,20,21,23,24] (3) Develop a health education plan [20]
4. Implementation plan	7	(1) Create a weight management profile [20] (2) Diet and exercise guidance [17,18,19,20,21,23,24] (3) Health education training [20,23,24] (4) Timely information support and psychological support [17,19,20,21,24]
5. Supervision and evaluation	5	(1) Periodic follow-up monitoring [15,18,19,20,24] (2) Evaluation every 3 to 6 months [20]
6. Adjust	1	Analyze the cause and adjust the cause [20]

## Data Availability

The original contributions presented in this study are included in the article. Further inquiries can be directed to the corresponding author.

## Appendix A

**Table A1:** Weight management protocols for patients with EC treated with fertility preservation.

**First-Level Entry**	**Second-Level Entry**	**Three-Level Entry**
1. Establishing the intervention development group	1.1 Membership composition	1.1.1 To establish a multidisciplinary weight management team for overweight/obese patients with EC treated with fertility preservation, with key members including gynecologists, endocrinologists, nutritionists, rehabilitation physicians, nurses, etc.
	1.2 Define responsibilities	1.2.1 Gynecologists are responsible for the treatment of patients with fertility preservation function, assessment of patients’ weight loss needs, and management of adverse reactions related to nursing therapy and nutritional interventions.
		1.2.2 Endocrinologists are responsible for the assessment of patients’ endocrine metabolism, such as thyroid and islet cell function, and the formulation of endocrine drug therapy to improve blood sugar and insulin resistance.
		1.2.3 Nutrition physicians are responsible for the nutritional assessment of patients, the formulation and adjustment of nutritional intervention programs, regular nutritional monitoring and effect evaluation.
		1.2.4 Rehabilitation physicians are responsible for the exercise assessment of patients, the formulation and adjustment of exercise intervention programs, regular exercise monitoring and effect evaluation.
		1.2.5 Nurses are responsible for overweight/obesity risk screening of patients, assessing patients’ willingness to lose weight, and establishing weight management files for patients; Assist gynecologists, endocrinologists, nutritionists and rehabilitation physicians in the evaluation and weight management program formulation, monitoring and effect evaluation; Follow up regularly, carry out health education, provide information and psychological support for patients, and participate in the whole process of weight management and guidance.
2. Screening evaluation	2.1 Screening	2.1.1 Timing: When it is clear that patients with EC have the intention of fertility preservation treatment, screening should be completed at the time of outpatient visit or admission.
		2.1.2 Contents: The patient’s height, weight, waist circumference and hip circumference were measured and BMI was calculated. BMI ≥ 24 kg/m^2^. Judged to be overweight; BMI ≥ 28 kg/m^2^ is considered obese, waist circumference ≥ 85 cm is considered central obesity.
	2.2 Evaluate	2.2.1 Basic information: age, marital status, living status, education level, family income, occupation and previous weight loss experience.
		2.2.2 Medical history: Family history, drug use history, smoking history, obesity-related comorbidities (type 2 diabetes, dyslipidemia, hypertension, coronary atherosclerotic heart disease, nonalcoholic fatty liver disease, sleep apnea syndrome, polycystic ovary syndrome, osteoarthritis, gout, etc.).
		2.2.3 Treatment of endometrial carcinoma or endometrial atypical hyperplasia.
		2.2.4 Willingness to lose weight.
		2.2.5 Body composition analysis, biochemical and metabolic indicators: triglyceride, total cholesterol, high density lipoprotein, low density lipoprotein, fasting blood glucose, fasting insulin, glycated hemoglobin, thyroid stimulating hormone, alanine transferase, blood creatinine, urea nitrogen, etc.
		2.2.6 Diet, exercise, lifestyle: Dietary nutrition Assessment questionnaire, physical activity assessment questionnaire, health promotion lifestyle scale were assessed.
		2.2.7 Health beliefs, self-efficacy, quality of life: The Health Belief Scale, self-efficacy scale, and General Quality of Life scale for cancer patients were evaluated.
	2.3 Judgment	2.3.1 Stage 1: Overweight with one or more overweight/obesity-related diseases; Or obese, without or with one or more overweight/obesity-related preconditions.
		2.3.2 Stage 2: Overweight/obesity with one or more overweight/obesity-related diseases.
		2.3.3 Stage 3: Overweight/obese with severe complications from one or more overweight/obesity-related diseases.
3. Make a plan	3.1 Set weight management goals	3.1.1 Those with normal weight at the first screening maintained their original weight.
		3.1.2 Weight loss of at least 5% within 3 to 6 months of initial screening.
		3.1.3 Those who were first screened for obesity lost at least 10% of their body weight within 3 to 6 months.
	3.2 Create personalized meal recipes	3.2.1 Dietary principles: less salt and less oil, control sugar and limit wine, reasonable collocation, balanced diet.
		3.2.2 Dietary structure: Overweight and obese people adopt a balanced diet with limited energy, that is, the daily distribution of the three major nutrients is carbohydrates accounted for 50% to 55%, protein accounted for 10% to 20%, fat accounted for 25% to 30%, and the total amount is reduced by 500 kcal on the basis of the target intake; Normal weight patients were instructed to eat a balanced diet and improve bad eating habits.
		3.2.3 Recipe formulation: The food exchange portion method was used as a guide, that is, each 90 Kcal calorie of food was taken as one portion, the ideal weight was calculated, the total daily intake of calories was calculated according to the activity intensity, the number of six food exchange portions and the distribution of three meals were determined according to the total calories and diet structure, and the specific recipes were formulated according to the tastes and preferences of patients combined with the equivalent food exchange table to ensure that patients could accept and implement them.
	3.3 Develop an individualized exercise prescription	3.3.1 Exercise principle: Take steps gradually, avoid sitting, and ensure at least 6000 steps a day.
		3.3.2 Total exercise: Overweight and obese people are recommended to consume 90 kcal calories as one exercise unit, daily exercise consumption of 3 to 4 exercise units; People of normal weight can be guided to develop their exercise habits.
		3.3.3 Type of exercise: such as dancing, aerobics, brisk walking and other light aerobic exercise, about 90 kcal every 20 min; Moderate aerobic exercise, such as climbing, jogging and badminton, consumes about 90 kcal every 10 min; Jumping rope, swimming, basketball and other heavy aerobic exercise consumes about 90 kcal every 5 min; Resistance exercises such as sit-ups, squats, dumbbell heel raises, etc.
		3.3.4 Exercise intensity: exercise 3~5 days a week, can exercise every day or every other day; During exercise, the heart rate ranges from 64% to 76%, and the maximum heart rate or exercise intensity is 3–6 exercise equivalent; Increase the intensity by 5% for every 6 sessions until you reach 65% of your maximum load.
		3.3.5 Formulation of prescription: According to the patient’s health status and personal preference, individual exercise prescription including exercise type, exercise intensity, exercise time, exercise frequency and other contents shall be formulated.
	3.4 Develop a health education plan	3.4.1 Time, form and frequency: Health education knowledge is promoted to patients through the online platform every two weeks, each time lasting 30 to 60 min, a total of 6 times.
		3.4.2 Contents: Adverse effects of overweight/obesity on treatment outcomes of fertility preservation; The benefits of diet and exercise for weight control; Difficulties and problems that may be encountered in weight control, and give solutions; Popularizing nutrition knowledge such as caloric estimation method and common nutrition misunderstanding; Sports knowledge popularization, such as sports precautions and sports methods; Peer experience sharing, etc.
4. Implementation plan	4.1 Put into administration	4.1.1 Add the patient to the WeChat group and keep timely communication and feedback.
		4.1.2 Establish a weight management profile for patients.
	4.2 Dietary program guidance	4.2.1 Time: The first time to communicate with the patient to develop a personalized diet plan.
		4.2.2 Contents: Use method and precautions of diet program.
	4.3 Exercise program guidance	4.3.1 Time: The first time to communicate with the patient to formulate an individualized exercise program.
		4.3.2 Content: Use method and precautions of exercise program.
	4.4 Health education and training	4.4.1 Time: After the patient started the diet and exercise program.
		4.4.2 Content: Same as health education program content.
	4.5 Timely information support and psychological support	4.5.1 Timely answer patients’ questions about diet, exercise, treatment and other aspects.
		4.5.2 Every day to the patient’s efforts to give affirmation, to the patient’s psychological dilemma to give resolution.
		4.5.3 Encourage patients to share diet and exercise records daily to promote peer support.
5. Supervision and evaluation	5.1 Weekly supervision	5.1.1 Supervise patients to make daily diet, exercise and weight records.
	5.2 Evaluation once every 3 months, at least 2 consecutive times	5.2.1 At 3 months, whether the weight management goal was achieved.
		5.2.2 Body mass index, waist circumference, body composition analysis, glycolipid metabolism index, etc.
		5.2.3 Remission of EC or endometrial atypical hyperplasia after treatment.
		5.2.4 Lifestyle, health beliefs, self-efficacy, quality of life, etc.
6. Analysis and adjustment	6.1 Analyze the reasons that hinder weight management	6.1.1 The diet and exercise program was adhered to well, but the weight control was not ideal: side effects of hormone therapy, insensitive diet and exercise program, etc.
		6.1.2 Diet and exercise program cannot be adhered to: Due to personal reasons such as changes in work and living environment or restrictions, the existing program cannot be adhered to.
		6.1.3 Other reasons.
	6.2 Adjust for cause	6.2.1 Change of endometrial treatment plan: Adjust to other treatment plan according to the pathological results of patients and drug side effects.
		6.2.2 Change of diet and exercise regimen: Consider the application of other diet or exercise regimen such as high-protein diet, intermittent energy restriction, ketogenic diet, etc.
		6.2.3 According to the patient’s personal situation, make the appropriate diet and exercise program fine-tuning.
